# Resveratrol Plays Protective Roles on Kidney of Uremic Rats via Activating HSP70 Expression

**DOI:** 10.1155/2020/2126748

**Published:** 2020-03-23

**Authors:** Shandan Feng, Jianjie Wang, Jian Teng, Zhan Fang, Chongting Lin

**Affiliations:** ^1^Department of Nephrology, Yantai Hospital of Traditional Chinese Medicine, Yantai, 264000 Shandong, China; ^2^Department of Nephrology, Yantaishan Hospital, Yantai, 264000 Shandong, China; ^3^Hemodialysis Room, Yantaishan Hospital, Yantai, 264000 Shandong, China

## Abstract

**Objective:**

To investigate the protective effects of resveratrol on kidney of uremic rats and to explore whether the mechanism is associated with heat shock protein 70 (HSP70) expression.

**Methods:**

Sixty male Sprague Dawley rats were randomly separated into 5 groups, including sham group, uremic model group, and different doses of resveratrol group (5 mg/kg, 10 mg/kg, and 20 mg/kg). The serum creatinine (Cr) and urea nitrogen (BUN) levels were detected by Automatic Biochemical Analyzer (ABA). The pathological changes of renal tissues and the renal interstitial fibrosis were analyzed by hematoxylin-eosin (HE) and Masson, respectively. The expression of HSP70 protein in renal tissues was detected by immunohistochemistry. The expression of HSP70 and NF-*κ*B pathway-related proteins were detected by Western blot. To further validate the protective role of resveratrol through activating HSP70 in uremic rats, HSP70 activator (17-AAG) and HSP70 inhibitor group (MKT-077) were used.

**Results:**

In the model group, the levels of Cr and BUN in serum were significantly increased, and the renal interstitial collagen deposition was also obviously increased (*p* < 0.05). Compared with the model group, the levels of Cr and BUN in different doses of resveratrol groups were remarkably declined, and the renal interstitial collagen deposition was declined (*p* < 0.05). Resveratrol also significantly improved the renal tissue lesions when compared with the model group. In renal tissues, different doses of resveratrol treatment remarkably raised HSP70 and p-I*κ*B*α* expression and also remarkably declined the level of p-P65 protein (*p* < 0.05). Meanwhile, the effect of 17-AAG was similar to 20 mg/kg resveratrol on NF-*κ*B pathway-related proteins expression. After the added MKT-077 in the resveratrol treatment group, the levels of HSP70 and p-I*κ*B*α* in the renal tissue were remarkably declined; however, the levels of p-P65 protein was remarkably raised (*p* < 0.05).

**Conclusion:**

Resveratrol played a protective role on the kidney of uremic rats through activating HSP70 expression.

## 1. Introduction

Uremia is a serious complication of kidney disease syndrome, which developed alongside the deterioration of renal function [1]. The most obvious feature is that uremia elevated concentrations of urea in the blood are involved with fluid, electrolyte, metabolic abnormalities, and hormone imbalances [2]. The number of uremic patients is ever-increasing with dysfunction of kidney; however, the treatments of uremic are still miserable. Worldwide, about 2 million people would die because of uremia [1]. To date, patients could keep alive by treating with kidney transplant and dialysis. But these people also suffer unbelievable pain with a higher rate of blood and heart problems [3].

Resveratrol (Res, 3,5,4′-trihydroxy-trans-stilbene) is a polyphenol which belongs to stilbenes family and widely exists in the related products of grape skin [4], such as berries and peanuts [5]. Various pharmacological effects of Res have been reported by previous studies, such as anticancer, anti-inflammatory, antidiabetic properties, antioxidant, and antiatherosclerotic [6–8]. Recent studies have examined the wide roles of Res in multiply organisms [5, 9], such as feeding rats with 3H-trans resveratrol resulted in the presence of Res in plasma [10]. Similarly, a single intravenous administration of trans-Res (15 mg/kg) in rats resulted in significantly raised in serum (0.025 *μ*M), in kidney (1.45 nmol/g), and lung (1.13 nmol/g) [11].

The heat shock protein 70 (HSP70) plays particular roles in the protection of kidney tissue. The extensive investigation of renal function and localization of HSP70 protein started from the 1990s [12]. HSP70 is the main constituent protein of HSP family, and it also expresses normally in all areas of renal tissue. Nuclear transcription factor-*κ*B(NF-*κ*B) is an essential transcriptional regulator of inflammation-related genes. Once activated, it would be triggering a declined cascade of inflammatory mediators, thereby, chiefly participating in the progression and onset of uremia [13]. In this study, the effect of resveratrol on kidney of uremic rats was studied, and the underlying role of resveratrol on HSP70 expression in kidney of uremic rats was also observed.

## 2. Materials and Methods

### 2.1. Experimental Animals

Sixty healthy male Sprague Dawley rats weighting 190-250 g aged 3 months were procured from Jinan Peng Yue Experimental Animal Breeding Co., Ltd. (permission number: SCXK (Shandong Province) 2014-0007, China). The rats were fed freely in a SPF laboratory with 22-24°C, 50-60% humidity. The animal study protocol was in line with the National Institutes of Health (NIH Pub. No. 85-23, revised 1996) Guide for the Care and Use of Laboratory Animals and confirmed by the Institutional Use Committee and Animal Care of Yantai Hospital of Traditional Chinese Medicine.

### 2.2. Uremic Rat Model

The uremic rat model was established through 5/6 nephrectomy [14]. In details, 12 hours of fasting were given for rats before operation. The rats were anesthetized with 40-50 mg/kg sodium pentobarbital by an intraperitoneal (IP) injection. Then, fixed the rats on the supine position and disinfected their surgical region of skin. Cutting the skin muscles from 1 cm under the left back rib to 0.5 cm away from the spine to find the left kidney and stripping the renal capsule, 1/3 of the upper and lower poles were trapezoidally resected. The left kidney was discarded 2/3 and given a gelatin sponge in incision site to stop bleeding, confirming no activity. After one week, the anesthesia method and position of the rats were the same as before, and the right kidney was taken after ligation of the renal pedicle at the right renal hilum. Penicillin was given for 3 days to prevent infection after the left and right nephrectomy.

### 2.3. Groups

Thirty rats were randomly separated into 5 groups:

1. Sham group: six rats were treated with opening the renal capsule and exposing the kidney for 5 min, then sutured the muscle layer and skin. After one week, the same amount of normal saline per day was intraperitoneally injected
2. Model group: six uremic rat models were established through 5/6 nephrectomy. After one week, the rats were treated with intraperitoneal injection of the same amount of normal saline per day
3. Low dose of resveratrol (Res-L) group: six uremic rat models were established through 5/6 nephrectomy, and after one week, 5 mg/kg resveratrol (suspended in 0.5% sodium carboxymethylcellulose) was intraperitoneally administered per day for 28 days
4. Medium dose of resveratrol (Res-M) group: six uremic rat models were established through 5/6 nephrectomy, and after one week, 10 mg/kg resveratrol (suspended in 0.5% sodium carboxymethylcellulose) was intraperitoneally administered per day for 28 days
5. High dose of resveratrol (Res-H) group: six uremic rat models were established through 5/6 nephrectomy, and after one week, 20 mg/kg resveratrol (suspended in 0.5% sodium carboxymethylcellulose) was intraperitoneally administered per day for 28 days

### 2.4. Sample Collection and Preparation

Following the last administration, blood specimens were gathered from the tail vein of each group, then centrifuged for 10 min at 3000 r/min. The supernatant was discarded. After anesthesia with 2% sodium pentobarbital (50 mg/kg), all rats were sacrificed using a cervical dislocation method. The kidney tissue of rats was taken and fixed part of it in 4% PBS paraformaldehyde and then embedded in paraffin for hematoxylin-eosin (HE) staining, Masson staining, and immunohistochemical detection. The remaining kidney specimens were stored in the liquid nitrogen then transferred to a -80°C refrigerator for Western blot detection.

### 2.5. Renal Function Test

The serum creatinine (Cr) and urea nitrogen (BUN) levels of rats were measured via an automatic biochemical analyzer to compare differences in renal function among different groups.

### 2.6. HE Staining

Tissue pieces (5 *μ*m) were successively deparaffinized with xylene and dehydrated in ascended alcohol series. The pieces were stained with hematoxylin (Solarbio, Beijing, China) for 5 min, then washed with water. After that, hydrochloric acid alcohol was used to differentiate pieces for 30 s and soaked into water for 15 min. The pieces were stained with eosin (Solarbio, Beijing, China) for 2 min. Finally, renal histopathological changes were scanned under the Olympus system Microscopes BX51 at 400 magnifications.

### 2.7. Masson Staining

These sections were routinely dewaxed, hydrated, stained with hematoxylin (Beijing Solarbio Science & Technology Co., Ltd., Beijing, China) for 5 min, and then separated in acidic ethanol differentiation solution. After adding Masson bluing solution, lichen red magenta dye solution was used for staining. After leaching with 2% glacial acetic acid solution, the sections were washed with phosphomolybdate solution for 3 min and subsequently stained with aniline blue staining solution for 5 min. The sections were dehydrated with 95% ethanol and anhydrous ethanol and permeabilized with xylene and sealed with neutral gum. The collagen in each group was examined under an optical microscope (Olympus Model BX51, Olympus, Japan). The percentage of collagen in each group was calculated using the ImageJ (NIH) software.

### 2.8. Immunohistochemical Assay

The paraffin-embedded slices were routinely sectioned and baked, deparaffinized with xylene, and hydrated with a gradient ethanol solution. The slices were then inactivated by soaking in a 3% H_2_O_2_ methanol solution for 20 min, heat-fixed with pH 6.0 citrate buffer for 10 min, and blocked using 5% BSA for 20 min. Rabbit anti-rat HSP70 (1 : 200, orb228105, Biorbyt, Cambridge, UK) polyclonal antibody was added and reacted overnight at 4°C. After rewarming, horseradish peroxidase-labeled goat anti-rabbit IgG (1 : 1000, ABIN101988, antibodies-online, Germany) was used for incubation with secondary antibody, then, incubated with SABC for 30 min, washed with PBS 3 times, colored with DAB, slightly restained with hematoxylin, dehydrated in ethanol gradient, cleared in dimethylbenzene, and mounted in neutral tree lipid. The findings were scanned under the Olympus system Microscopes BX51 at 400 magnifications and analyzed by the Aperio Imagescope 11.1 software.

### 2.9. Western Blotting

Kidney tissues were ground and homogenized and centrifuged at 10,000 rpm about 10-30 min. The supernatant was taken for the next experiments. The protein concentration was measured by the BCA kit (Solarbio, Beijing, China). 40 *μ*g of the protein sample was mixed with 10% SDS gel buffer with a ratio of 1 : 1. The protein was denatured by heating at 95°C for 5 min. The PVDF membrane (Merck, Darmstadt, Germany) was rotated at 80 V for 30 min. It was then blocked with 5% skim milk powder in TBST for 1 h at 4°C. Next, TBST solution containing 3% bovine serum albumin was used to dilute HSP70 (1 : 500, orb228105), P65 (1 : 500, orb229138), p-P65 (1 : 500, orb304662), I*κ*B*α* (1 : 500, orb223182), p-I*κ*B*α* (1 : 500, orb223035), and *β*-actin (1 : 2000, orb178392) (all Biorbyt Ltd., Cambridge, UK). The reaction was remained overnight at 4°C. Prior to testing, the antibody was rewarmed and then incubated with horseradish peroxidase-labeled goat anti-rabbit IgG (1 : 1000, ABIN101988, antibodies-online, Aachen, Germany) for 1 h. It was washed with ECL luminescent substrate for 3-5 min. The expression levels of proteins were normalized by *β*-actin.

## 3. Elisa

Serum tumor necrosis factor *α* (TNF-*α*) (orb79138-480), Interleukin-1 *β* (IL-1*β*) (orb79117), IL-6 (orb79123), and IL-8 (orb312288) were detected in strict line with the ELISA kit instructions (all Biorbyt Ltd., Cambridge, UK). The optical densities at 450 nm were assessed with a microplate reader (RT-6100, Lei Du).

### 3.1. Validate the Protective Role of Resveratrol through Activating HSP70 in Uremic Rats

In this study, the effect of the high dose of resveratrol on kidney was obvious, so we chose the high dose of resveratrol (20 mg/kg) for further experiments. Thirty rats were haphazardly separated into 5 groups, 6 each group: sham group (Sham), model group (Model), resveratrol group (Res) (after modeling for 1 week, the rats were intraperitoneally injected with 20 mg/kg resveratrol for 28 days), HSP70 activator group (17-AAG) (after modeling for 1 week, the rats were intraperitoneally injected with 17-AAG 80 mg/kg per day for continuous 28 days [15]), and resveratrol+HSP70 inhibitor (MKT-077) group (Res+MKT) (one week after modeling, 20 mg/kg resveratrol was injected intraperitoneally and 5 mg/kg MKT-077 was intravenously injected, with continuous administration for 28 days [16]).

### 3.2. Statistical Analysis

All statics in the present study were analyzed by the SPSS 19.0 software. Mean ± SD was used to measure the categorical data. The data was analyzed by standard one-way ANOVA, then using Tukey's test between groups. *p* < 0.05 was considered as statistically significant.

## 4. Results

### 4.1. Effects of Resveratrol on Serum Cr and BUN in Uremic Rats

**Figure 1:**
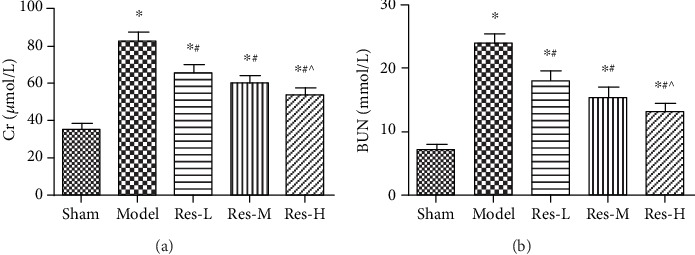
Effects of resveratrol on serum Cr (a) and BUN (b) levels in uremic rats. Compared to the sham group, ^∗^*p* < 0.05; contrasted to the model group, #*p* < 0.05; contrasted to the Res-L group, ^*p* < 0.05.

The blood specimens were collected for subsequent statistical analysis. As we all know, the high levels of serum Cr and BUN in blood usually means the failure of renal tissue. The results showed in Figure 1 have revealed that the levels of serum Cr and BUN in the model group were remarkably increased compared with the sham group (*p* < 0.05). But in the Res groups, the levels of serum Cr and BUN obviously decreased and contrasted to the model group (*p* < 0.05). Moreover, the concentration of resveratrol influenced the impairment of kidney tissue. These results suggested the protection role of resveratrol on kidney tissue in uremic rats.

### 4.2. Effects of Resveratrol on Histology of Kidney in Uremic Rats

**Figure 2:**
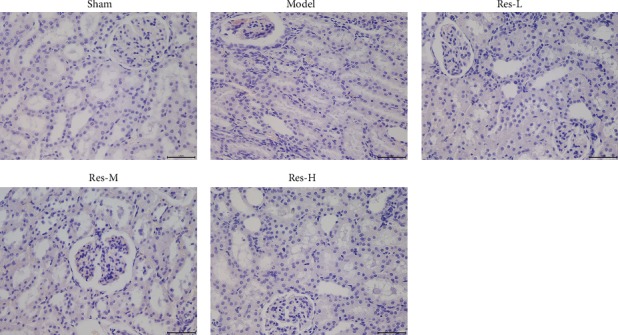
Effects of resveratrol on kidney tissue histopathology in uremic rats. Scale bar = 50 *μ*m.

The HE staining images in Figure 2 displayed that the sham group had normal renal interstitial, renal glomerular, and tubular morphologies, whereas the model group underwent evident glomerular vascular disordering, the cyst wall thickening, luminal narrowing, and obvious inflammatory cell infiltrating. However, the renal lesions were remarkably managed, and the inflammatory response was reduced by different doses of resveratrol treatment. Furthermore, pathological damage was alleviated to a great extent in the Res-H group compared to the Res-L group. Obviously, these results indicated that resveratrol also enhanced the injury of kidneys in uremic rats.

### 4.3. Effects of Resveratrol on Renal Interstitial Fibrosis in Uremic Rats

**Figure 3:**
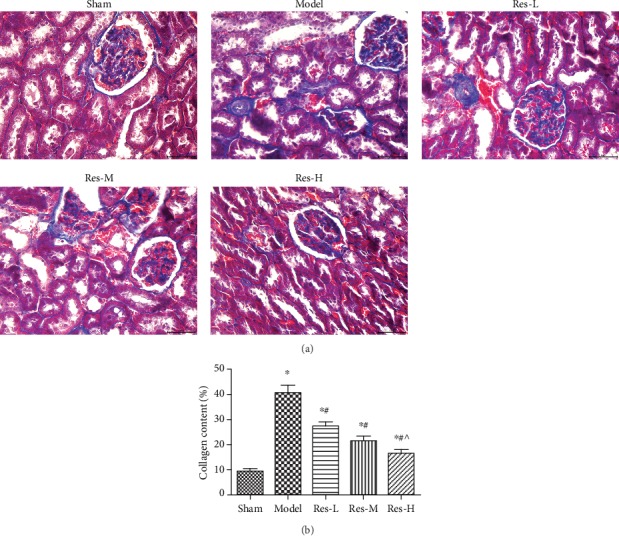
Effects of resveratrol on renal interstitial fibrosis in kidney of uremic rats. (a) Renal tissue was colored red and collagen was colored blue by Masson staining, Scale bar = 50 *μ*m. (b) Collagen content was analyzed by the ImageJ software. Contrasted to the sham group, ^∗^*p* < 0.05; contrasted to the model group, #*p* < 0.05; contrasted to the Res-L group, ^*p* < 0.05.

To investigate the impact of resveratrol on renal interstitial fibrosis in uremic rat, Masson staining was used to detect the renal interstitial fibrosis (Figure 3). In the model group, the arrangement of collagen fibers was in poor order, and the amount of renal interstitial collagen was remarkably raised compared with the sham group (*p* < 0.05). In different doses of resveratrol group, the deposition of renal interstitial collagen was remarkably declined with comparison to the model group (*p* < 0.05). Moreover, the concentration of resveratrol influenced the renal interstitial fibrosis. These results suggested that resveratrol improved renal interstitial fibrosis in uremic rats.

### 4.4. Effects of Resveratrol on Hsp70 Protein Expression in Kidney Tissues of Uremic Rats

**Figure 4:**
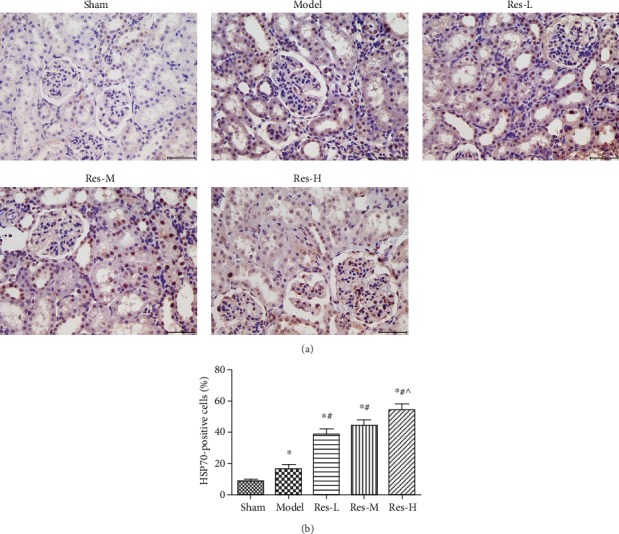
Effects of resveratrol on Hsp70 protein expression in kidney tissues of uremic rats. (a) The Hsp70 protein expression was measured by immunohistochemical staining, Scale bar = 50 *μ*m. (b) Percentage of Hsp70 positive expressions was analyzed by the ImageJ software. Contrasted to the Sham group, ^∗^*p* < 0.05; contrasted to the model group, #*p* < 0.05; contrasted to the Res-L group, ^*p* < 0.05.

As shown in Figure 4, the expression of Hsp70 protein in the model group was significantly lower than that in the sham group (*p* < 0.05). Compared to the model group, resveratrol treatments markedly elevated the abundance of Hsp70 in kidney tissue (*p* < 0.05). We also found that the expression of Hsp70 protein was statistically associated with the concentration of resveratrol.

### 4.5. Effects of Resveratrol on the Expression of Related Proteins in Renal Tissues of Uremic Rats

**Figure 5:**
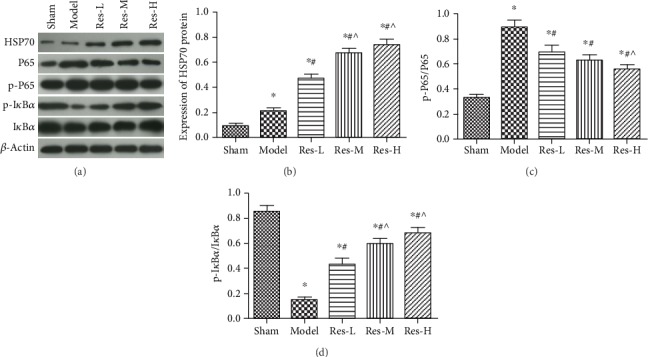
Effect of resveratrol on the expression of HSP70 and NF-*κ*B signaling pathway-related proteins in renal tissues of uremic rats. (a) Protein bands, *β*-actin was referred to internal reference; (b) the relative expression of HSP70; (c) the relative expression of p-P65/P65; (d) the relative expression of p-I*κ*B*α*/I*κ*B*α*. Contrasted to the sham group, ^∗^*p* < 0.05; contrasted to the model group, #*p* < 0.05; contrasted to the Res-L group, ^*p* < 0.05.

To demonstrate the linkage between resveratrol and NF-*κ*B signaling pathway, Western blot was used to examine the expression of Hsp70, phosphorylated I*κ*B*α* (p-I*κ*B*α*), and NF-*κ*B p65 (p-P65). As shown in Figure 5, the expression of Hsp70 and p-I*κ*B*α* protein in the model group were remarkably declined; however, the expression of p-P65 protein was remarkably raised compared with the sham group (*p* < 0.05). Compared with the model group, the expression of Hsp70 and p-I*κ*B*α* protein were remarkably raised; yet, the expression of p-P65 protein was remarkably declined after the dose of resveratrol treatment (*p* < 0.05). The significant changes existed between Res-H group and Res-L group (*p* < 0.05).

### 4.6. Effects of Resveratrol on Serum IL-6, IL-8, TNF-*α*, and IL-1*β* Levels in Uremic Rats

**Figure 6:**
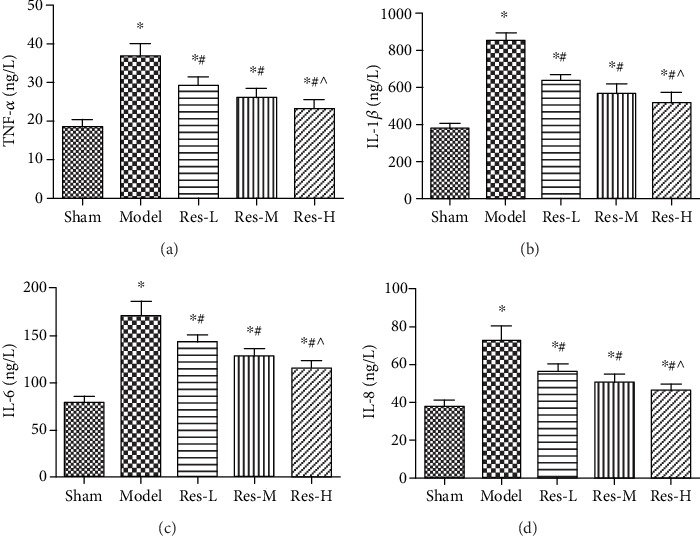
Effect of resveratrol on serum IL-6, IL-8, IL-1*β*, and TNF-*α* levels in uremic rats. (a) TNF-*α*, (b) IL-1*β*, (c) IL-6, and (d) IL-8. Contrasted to the sham group, ^∗^*p* < 0.05; contrasted to the model group, #*p* < 0.05; contrasted to the Res-L group, ^*p* < 0.05.

ELISA was performed to evaluate the serum IL-6, IL-8, TNF-*α*, and, IL-1*β* levels in the experimental groups (Figure 6). After surgery, the serum IL-6, IL-8, TNF-*α*, and IL-1*β* levels were increased in uremic rats with comparison to the sham group (*p* < 0.05). Compared to the model group, the expression of IL-6, IL-8, TNF-*α*, and IL-1*β* was declined after resveratrol treatment (*p* < 0.05). And the levels in Res-H group was remarkably lower than that in Res-L group (*p* < 0.05). These data suggested that the lower level of IL-6, IL-8, TNF-*α*, and IL-1*β* were correlated with the concentration of resveratrol in uremic rats.

### 4.7. Resveratrol Activates HSP70 Expression to Regulate NF-*κ*B Signaling Pathway

**Figure 7:**
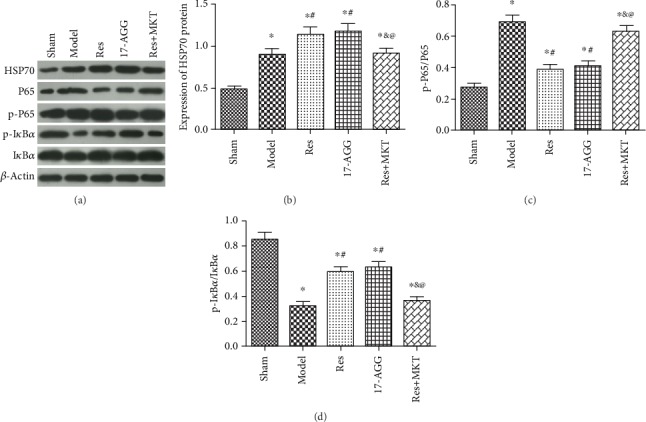
Resveratrol-activated HSP70 expression to regulate NF-*κ*B pathway-related proteins expression. (a) Protein bands, *β*-actin was referred to internal reference; (b) the relative expression of HSP70; (c) the relative expression of p-P65/P65; (d) the relative expression of p-I*κ*B*α*/I*κ*B*α*. Contrasted to the sham group, ^∗^*p* < 0.05; contrasted to the model group, #*p* < 0.05; contrasted to the Res group, ^&^*p* < 0.05; contrasted to the 17-AAG group, ^@^*p* < 0.05.

To further validate, resveratrol plays effects on NF-*κ*B signal pathway via activating the expression of Hsp70 protein (Figure 7). 17-AAG, as the HSP70 activator, was injected. MKT-077, as the HSP70 inhibitor, was injected after resveratrol administration. Contrasted to the model group, the protein levels of HSP70 and p-I*κ*B*α* were remarkably raised, and the expression of p-P65 protein was remarkably declined in the renal tissues of Res and 17-AAG groups (*p* < 0.05). Compared to Res and 17-AGG groups, the levels of HSP70 and p-I*κ*B*α* protein were remarkably declined, and the expression of p65 protein was remarkably elevated in the renal tissue of Res+MKT group (*p* < 0.05). Among these results, we deduced that resveratrol activated Hsp70 expression to regulate NF-*κ*B signaling pathway in kidney of uremic rats.

## 5. Discussion

Uremia is a common health problem, which always developed with chronic kidney disease (CKD), particularly in the later development stages of CKD [1, 17, 18]. Moreover, it may cause the rapid organ dysfunction syndrome of renal tissue and eventually result in death [19]. To date, the role of resveratrol in kidney-related diseases had been explored by various studies. In mice, however, it has been shown that resveratrol treatment inhibits oxidative stress and renal interstitial fibrosis [20]. A previous report showed that resveratrol played protective effects on septic kidney injury rats [5]. But the underlying mechanism of resveratrol is remaining unknown. In the present study, we first constructed a rat model of uremic through 5/6 nephrectomy to investigate the effects of resveratrol on uremic kidney rats [9]. Our results were consistent with previous studies, that resveratrol administration significantly ameliorated the damages of renal tissue in uremic rats.

In this study, the expression of HSP70 protein was remarkably raised in renal tissues after resveratrol treatment. It meant that resveratrol treating could regulate to the expression of Hsp70 in kidney of uremic rats. As the same time, resveratrol treatments changed the NF-*κ*B signaling-related proteins. P65 protein is a major subunit of the NF-*κ*B signaling pathway [10, 21]. Previous evidences showed that the protein levels of p-P65 and P50 were upregulated, and the activation of NF-*κ*B signaling pathway was enhanced in inflammatory kidney disease [22–25]. Consistent with another study, administration with resveratrol significantly expressed the p-I*κ*B*α* subunit in animals with CKD [26]. The data in this research showed that p-I*κ*B*α* protein was remarkably raised, yet the expression of p-P65 protein was remarkably declined after the dose of resveratrol treatment, when compared to model group.

In order to confirm that resveratrol activates Hsp70 expression to regulate NF-*κ*B signaling pathway in kidney of uremic rats, the HSP70 activator (17-AAG) and inhibitor (MKT-077) were injected. The results showed that the effect of 17-AAG was similar to the 20 mg/kg resveratrol on NF-*κ*B pathway-related proteins expression. Adding MKT-077, the effect of resveratrol on NF-*κ*B pathway-related proteins was oppositely changed.

## 6. Conclusion

In summary, we found that resveratrol treatment played the protective effects on kidney of uremic rats and regulated NF-*κ*B pathway-related proteins expression via activating HSP70 expression. In the future, more studies about uremic disease will be designed to find more suitable methods for rescuing the uremic patients.

## Data Availability

The analyzed data sets generated during the study are available from the corresponding author on reasonable request.
